# A Neuropsychiatric Prelude to Unveiling Small Cell Lung Cancer with Suspected Paraneoplastic Limbic Encephalitis: A Case Report

**DOI:** 10.3390/curroncol32060366

**Published:** 2025-06-19

**Authors:** Jessa Letargo, X. Melody Qu, Timothy K. Nguyen, Alexander V. Louie, Sara Kuruvilla, Enxhi Kotrri

**Affiliations:** 1 Schulich School of Medicine & Dentistry, Western University, London, ON N6A 5C1, Canada; 2 Division of Radiation Oncology, London Health Sciences Centre, Western University, London, ON N6A 5W9, Canada; melody.qu@lhsc.on.ca (X.M.Q.);; 3 Department of Radiation Oncology, Sunnybrook Health Sciences Centre, University of Toronto, Toronto, ON N6A 5W9, Canada; 4 Division of Medical Oncology, London Health Sciences Centre, Western University, London, ON N6A 5W9, Canada

**Keywords:** small cell lung cancer, paraneoplastic, limbic encephalitis

## Abstract

Small cell lung cancer (SCLC) is an aggressive form of lung cancer characterized by rapid growth and early metastases. As a neuroendocrine tumour, SCLC is especially notorious for various paraneoplastic syndromes, one of which is a rare neurological syndrome called paraneoplastic limbic encephalitis (PLE) that manifests with amnestic cognitive impairment and seizures. Here, we describe a case of a 53-year-old female who presented with neuropsychiatric symptoms of delusions, hallucinations, and cognitive impairment that started months prior to being diagnosed with extensive-stage SCLC. With no previous neuropsychiatric history, this raised the question of whether her presentation was related to PLE rather than a primary psychiatric condition, as initially diagnosed. Her symptoms improved with chemotherapy and radiation treatment of the underlying cancer, favouring a paraneoplastic etiology. Overall, this case underscores the importance of considering paraneoplastic syndromes in patients presenting with new neuropsychiatric symptoms, as early recognition and treatment can improve prognosis.

## Simple Summary

Paraneoplastic limbic encephalitis is a rare manifestation of malignancies such as small cell lung cancer, which can precede the cancer diagnosis and mimic other conditions. Early recognition of paraneoplastic neurological syndromes in patients with unexplained neuropsychiatric symptoms may lead to more timely cancer diagnosis, treatment, and improved outcomes.

## 1. Introduction

Lung cancer remains the leading cause of cancer-related mortality worldwide, with nearly 1.8 million deaths annually [1]. Small cell lung cancer (SCLC) accounts for approximately 15% of all bronchogenic cancers, and is a highly aggressive neuroendocrine carcinoma that is characterized by rapid tumour proliferation and early metastatic potential [2,3,4,5]. The staging of SCLC is classified as limited stage, in which the entire disease is confined to one hemithorax, or extensive stage, in which the disease is more widespread, extending beyond the primary hemithorax and/or including distant metastases [4]. Two-thirds of SCLC patients are diagnosed with extensive-stage disease on presentation.

Paraneoplastic syndromes are a heterogeneous group of syndromes that affect up to 8% of cancer patients, most commonly patients with SCLC [6,7]. Paraneoplastic limbic encephalitis (PLE) is one such rare paraneoplastic neurological syndrome driven by the host’s autoimmune response to tumour-expressed onconeural antigens, which mimic those naturally found in the limbic system [6,8]. This results in the production of onconeural antibodies (e.g., anti-Hu), which mounts a T-cell-mediated attack against the host’s limbic system, resulting in limbic inflammation and dysfunction that clinically manifest as neuropsychiatric symptoms such as seizures, confusion, mood changes, hallucinations, and memory loss [9].

Here, we present a case of a 53-year old woman who initially presented with neuropsychiatric symptoms months prior to being diagnosed with SCLC, which poses the question of whether this was PLE preceding the malignancy diagnosis.

## 2. Case Description

A 53-year-old female with a remote 20 pack-year smoking history, but otherwise healthy with no significant medical history, presented to the emergency department (ED) in October 2022 with a 6-day history of distressing neuropsychiatric symptoms, including hallucinations and delusions surrounding a fear of mice, memory dysfunction, sensory disturbances, and headaches. There were no known inciting events such as a history of falls, seizures, or head trauma. She did not have a history of any psychiatric or neurological conditions. Her Glasgow Coma Scale score was 15 on presentation, but mental status assessment revealed evidence of psychomotor agitation, forced speech, and partial insight and judgement. She was hemodynamically stable, and the rest of her physical exam was unremarkable, although a thorough neurologic examination was not documented.

She was admitted to hospital for further investigation, and underwent an initial computed tomography (CT) scan of the head, which did not show any abnormalities. Subsequent magnetic resonance imaging (MRI) of the brain showed an incidental finding of a small right frontal lobe meningioma measuring 1.1 × 0.4 cm, without mass effect and non-contributory to her presentation. There was no mesial temporal signal change. Her bloodwork was also unremarkable, with normal complete blood count, electrolytes, and thyroid-stimulating hormone, as well as a negative beta-human chorionic gonadotropin (b-hCG) and urine toxicology screen. She completed cognitive testing using the Montreal Cognitive Assessment (MoCA) tool, which yielded a score of 25/30 on two different occasions, indicating mild cognitive impairment (MCI). Her presentation was thought to be in keeping with delusional disorder and she was started on antipsychotics, which reportedly improved some of her presenting symptoms, but not back to her baseline. She was discharged after a month-long admission with outpatient psychiatry follow-up and a referral to neurology to rule out an underlying temporal seizure.

**Figure 1:**
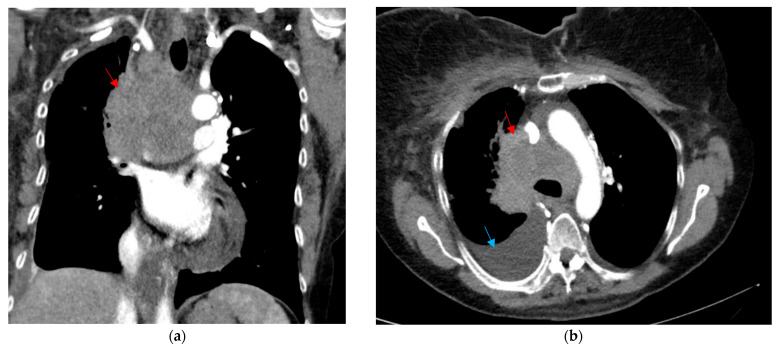
CT thorax shown in the (**a**) coronal soft tissue window and (**b**) axial soft tissue window, depicting a large mediastinal and right hilar mass measuring 7.5 × 3.6 cm, surrounding the posterior aspect of the superior vena cava (red arrow). There is also a moderate-sized right pleural effusion that is visualized (blue arrow).

Before being assessed by outpatient neurology, she presented again to the ED in January 2023 with a 1-week history of facial edema and dizziness. She was not endorsing any chest pain, dyspnea, cough, hemoptysis, or stridor at this time. A CT scan of the chest showed a large 7.5 × 3.6 cm conglomerate mass involving the mediastinum and right hilum, causing compression of the superior vena cava (SVC syndrome) (Figure 1). There were also bilateral pleural effusions, more pronounced on the right.

She underwent endoscopic ultrasound of the esophagus (EUS), as well as fine needle aspiration biopsy (FNAB) of the mediastinal mass and a right thoracentesis for pleural fluid cytology, both of which returned positive for small cell carcinoma. Staging scans included a CT of the abdomen and pelvis, which identified a concerning small liver lesion; however, repeat scans showed this to more likely be a hemangioma, and there were no other concerning findings for metastatic disease. An MR of the head was also clear for intracranial metastasis. A bone scan showed no evidence of osseus metastasis. Given the biopsy-proven evidence of metastasis to the pleural fluid, she was ultimately diagnosed with extensive-stage SCLC.

She was then assessed by Neuro-Oncology, whose neurological examination revealed slowed verbal responses, two-word and five-word 5-minute delayed recall, and normal strength testing in the upper and lower extremities, with some evidence of give-way weakness. From their assessment, Neuro-Oncology suspected mesial temporal lobe seizures, which could explain the remote phantom smell of rodents that she had endorsed several months prior. By this time, her hallucinations, delusions, and confusion had reportedly improved, but she continued to have objective memory dysfunction. With her prior psychiatric admission, and the newly diagnosed SCLC, there was a high suspicion for PLE. As such, a paraneoplastic serum panel was ordered, which yielded negative results (Table 1). In consideration of the patient’s frailty and poor Eastern Cooperative Oncology Group (ECOG) performance status of 3, a lumbar puncture was not performed at the time. Similarly, an electroencephalogram (EEG) was not pursued, as it was deemed unlikely to alter the management plan, given that immunosuppressive therapy was not considered appropriate due to her poor performance status. It was thought that treatment of the underlying cancer would also manage the suspected paraneoplastic neurological syndrome.

Given the complexity of her presentation, poor functional status, and progressive symptoms, including the development of hemoptysis, her case was discussed at a multidisciplinary cancer conference. Due to the urgent nature of super vena cava obstruction, and the uncertainty of the chemotherapy start date, the decision was made to start radiation treatment to the mediastinal and right hilar disease, with a planned dose of 20 Gray (Gy) in five fractions (fx). She received one fraction before her radiation was discontinued, as she started on systemic therapy with cisplatin, etoposide, and durvalumab the following day. In addition to a thoracentesis, her treatment resulted in a significant improvement in her respiratory symptoms. Her psychiatric symptoms also improved after starting systemic treatment, which allowed for most of her antipsychotic medications to be discontinued after the first cycle of chemotherapy. She was maintained on valproic acid for the suspected temporal seizures.

**Figure 2:**
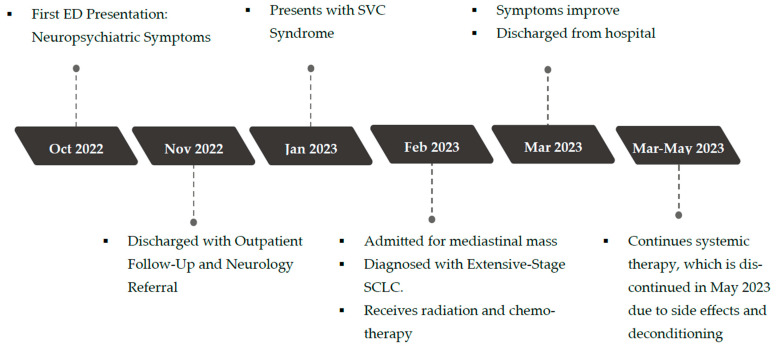

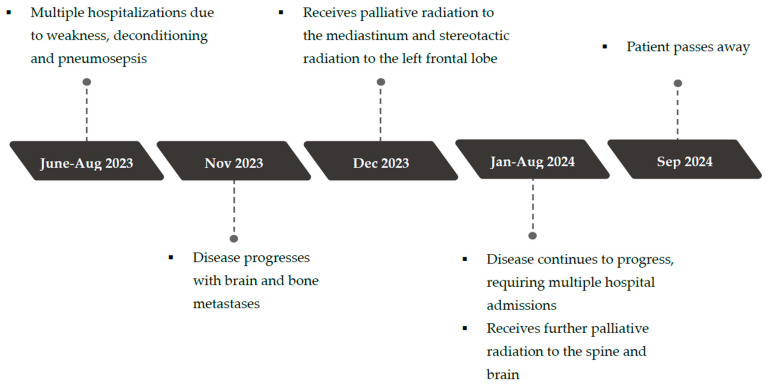
Timeline of events.

Her disease eventually progressed, with the development of brain and bone metastases. She ultimately succumbed to her disease in September 2024. A timeline of the clinical events over a two-year period from presentation to end-of-life is depicted in Figure 2.

## 3. Discussion

Paraneoplastic limbic encephalitis is an immune-mediated inflammatory syndrome of the limbic system caused by an underlying malignancy, most commonly associated with lung cancer [10]. The limbic system is a complex set of neural structures—including the hippocampus, cingulate gyrus, and amygdala—responsible for memory formation, behaviour, emotions, and olfactory associative learning [11]. Therefore, PLE can manifest clinically as disturbances in any of these domains, although other areas of the nervous system can also be involved [12]. There are many cases of PLE reported in the literature, with various presentations including the subacute onset of memory loss, confusion, seizures, anxiety, agitation, and personality changes [13,14,15,16,17,18,19,20,21]. Here, the patient similarly presented with confusion, agitation, phantom smells, hallucinations, and sensory disturbances, resulting in great fear and anxiety a few months before her diagnosis with small cell lung cancer. Her neuropsychiatric symptoms were concordant with disturbances to the limbic system, and the SCLC diagnosis shortly thereafter posed suspicion for PLE. Her neuropsychiatric condition improved after the first cycles of chemotherapy, but persisted a few months into her treatment. Nevertheless, her neuropsychiatric symptoms did not recur after her SCLC progressed at a later stage.

PLE can be challenging to recognize, as it often precedes the diagnosis of the malignancy by an average of 3.5 months, and its clinical presentation can mimic other neurological or psychiatric disorders such as dementia, delirium, infectious encephalitis, and other metabolic encephalopathies [6,9,12,22]. To address the challenges in diagnosing PLE, a group of neurologists, Graus et al., proposed four diagnostic criteria: (1) subacute onset (less than 3 months) of memory deficits, seizures, or psychiatric symptoms; (2) bilateral brain abnormalities in the medial temporal lobes shown on T2-weighted MRI; (3) at least one of the following: CSF pleocytosis or EEG with epileptic or slow-wave activity of the temporal lobes; and (4) reasonable exclusion of alternative causes [23]. While it is certainly ideal to meet all four criteria to confirm the diagnosis of PLE, in clinical practice, the logistical limitations, resource constraints, and invasiveness of the required tests are often difficult to balance with optimal patient care and quick disease progression. In this case, the patient did not undergo a lumbar puncture or an EEG. Her T2-weighted MR head did not show any temporal lobe abnormalities either. As a result, she does not meet Graus 2016 criteria for autoimmune limbic encephalitis. Still, suspicion for PLE remained high due to the subacute onset of neuropsychiatric dysfunction, which preceded the diagnosis of SCLC by approximately three months (Figure 2), as well as the presence of suspected temporal lobe seizures that were likely managed by the valproic acid initially prescribed to stabilize her mood. Additionally, the patient demonstrated neurologic deficits on examination, including slowed verbal responses, delayed recall, and weakness in strength testing. Her presentation could not be explained by alternative etiologies such as infectious, septic, or metabolic encephalopathy, drug toxicity, or autoimmune conditions, given her unremarkable bloodwork and neuroimaging. In recognizing the limitations in establishing a definitive diagnosis of PLE, Graus et al. proposed another set of diagnostic criteria for possible autoimmune limbic encephalitis [23], shown in Table 2, which fit this patient’s overall clinical picture (fulfilling criteria 1, 2a, 2b, and 3).

Antibody testing can aid in the diagnosis of PLE. The discovery of onconeural antibodies was revolutionary in confirming the paraneoplastic nature of the disease. The most common antibodies associated with SCLC and PLE are anti-Hu and anti-Ma2, followed by anti-CRMP5, anti- GABA_B_R, anti-PCA2, anti-AMPAR, and anti-GAD [24,25,26]. The presence of these antibodies is not only helpful in supporting an autoimmune etiology, but can also trigger a malignancy screening in patients not known to have an underlying cancer, guide treatment, and prognosticate the disease [9,25]. In a case report of a 59-year-old female who presented with seizures, memory changes, and decreased concentration, a prompt diagnosis of autoimmune limbic encephalitis was established via positive anti-Hu testing and neuroimaging. This led to a malignancy work up, which confirmed small cell carcinoma, allowing for prompt treatment of both the malignancy with systemic therapy, and the encephalitis with IV immunoglobulin and methylprednisolone [18].

However, there are certainly limitations in antibody testing, as it may not be easily accessible and results often take several weeks to obtain [23]. As such, it is advised not to be over-reliant on antibodies to make a prompt diagnosis of PLE [9]. Furthermore, the absence of autoantibodies does not automatically exclude the diagnosis of PLE. In another case report, a 42-year-old female was diagnosed with seronegative autoimmune limbic encephalitis after presenting with fever, headaches, memory deficits, and focal seizures. Investigations revealed bilateral temporal abnormalities on brain MRI and temporal seizures on EEG, but lumbar puncture showed CSF pleocytosis with negative serological testing for acute infections and negative onconeural antibodies [19]. It is estimated that approximately 7–26% of reported autoimmune limbic encephalitis cases have no detectable antibodies [19,27].

With the improved understanding of PLE, it is increasingly becoming more recognized in the differentials for the subacute onset of neuropsychiatric symptoms. However, it remains a complicated condition due to its rarity and wide constellation of presenting symptoms, thereby leading to delayed diagnosis and management.

## 4. Conclusions

Overall, this case highlights the importance of having a high index of suspicion for PLE and underlying malignancy such as SCLC in a patient who presents with otherwise unexplained neuropsychiatric symptoms and a significant smoking history. With the aggressive nature of SCLC, early and accurate diagnosis is crucial for the timely initiation of optimal treatment and improved patient outcomes. This case also underscores the importance of a multidisciplinary approach in the diagnosis and treatment of PLE-associated SCLC, as well as the need for continued research and education to overcome the diagnostic challenges in the early recognition of PLE, especially in patients with atypical presentation or no known malignancy.

## Figures and Tables

**Table 1:** Paraneoplastic serum panel.

Anti-AMPAR1/R2 CBA	Negative
Anti-CASPR2 CBA	Negative
Anti-DPPX CBA	Negative
Anti-GABARB1/B2 CBA	Negative
Anti-LGI1 CBA	Negative
Anti-NMDAR CBA	Negative
Anti-Amphiphysin Immunoblot	Negative
Anti-GAD65 Immunoblot	Negative
Anti-Hu Immunoblot	Negative
Anti-Ma2:Ta Immunoblot	Negative
Anti-Recoverin Immunoblot	Negative
Anti-Ri Immunoblot	Negative
Anti-SOX1 Immunoblot	Negative
Anti-Titin Immunoblot	Negative
Anti-Tr (DNER) Immunoblot	Negative
Anti-Yo Immunoblot	Negative
Anti-Zic4 Immunoblot	Negative
Anti-GAD65 ELISA	<5

**Table 2:** Diagnostic criteria for possible autoimmune limbic encephalitis (proposed by Graus et al.).

1	Subacute onset (rapid progression of less than 3 months) of working memory deficits (short-term memory loss), altered mental status, or psychiatric symptoms.	
2	At least one of:	a. New focal CNS findings
		b. Seizures not explained by a previously known seizure disorder
		c. CSF Pleocytosis (WBC > 5 cells mm^3^)
		d. MRI features suggestive of encephalitis
3	Reasonable exclusion of alternative causes	

## Data Availability

No new data were created or analyzed in this study.
